# Critical role for platelet Ral GTPases in regulating venous thrombosis in mice

**DOI:** 10.1016/j.jtha.2024.10.010

**Published:** 2024-10-24

**Authors:** Yong Li, Jonathan A. Furniss, Jordan Vautrinot, Christopher M. Williams, Tony G. Walsh, Alexander Brill, Borko Amulic, Alastair W. Poole

**Affiliations:** 1 School of Physiology, Pharmacology & Neuroscience, University of Bristol, Bristol, United Kingdom; 2 Institute of Cardiovascular Sciences, College of Medical and Dental Sciences, University of Birmingham, Birmingham, United Kingdom; 3 School of Cellular and Molecular Medicine, University of Bristol, Bristol, United Kingdom

**Keywords:** NET, P-selectin, RalA, RalB, venous thrombosis

## Abstract

**Background:**

Deep vein thrombosis is a major cause of morbidity and mortality globally. While its pathophysiology is complex, increasing evidence suggests a more prominent role for platelets than previously suspected. Genetic deletion of Ral GTPases, RalA and RalB, conditionally in mouse platelets (RalAB double knockout [DKO]), results in a near complete defect in P-selectin externalization upon activation, while other platelet activation responses and arterial thrombosis are preserved.

**Objectives:**

Given the critical role of P-selectin in mediating platelet-neutrophil interaction and thromboinflammation, we sought to investigate whether platelet Rals would also play critical roles in venous thrombosis, a thromboinflammatory disease, using RalAB DKO mice.

**Methods:**

Deep vein thrombosis was induced by surgical partial ligation of the caudal (inferior) vena cava for 24 hours or 48 hours before venous thrombi were assessed by histology and immunofluorescence microscopy.

**Results:**

RalAB DKO mice showed a reduction in venous thrombus formation after 24 hours and near complete ablation of venous thrombosis by 48 hours post inferior vena cava ligation. Immunofluorescence microscopy revealed that cross-sections of thrombi from wild-type mice consisted of an organized scaffolded structure of platelets surrounding leukocytes/neutrophils producing neutrophil extracellular traps (NETs) to stabilize and propagate the thrombus. This organized structure was absent in platelet-specific conditional RalAB DKO thrombi. *In vitro* analysis of platelet-mediated NET formation was also significantly reduced when platelets lacked RalAB or when platelets were treated with the Ral inhibitor RBC8.

**Conclusion:**

We identify platelet Rals as novel, potentially critical regulators of venous thrombus stability through their ability to regulate neutrophil NET formation via platelet P-selectin.

## 1 Introduction

Venous thromboembolism, including deep vein thrombosis (DVT) and pulmonary embolism, is a major source of morbidity and mortality worldwide, whose prevalence is greatest among patients with cancer, major trauma, patients undergoing surgery, or those with extended periods of immobilization [1–3]. Experimental *in vivo* models of DVT implicate a complex interplay between activated endothelium (P-selectin and von Willebrand factor [VWF]), platelets (P-selectin, glycoprotein [GP] Ibα, and high-mobility group box 1), and innate immune cells (neutrophil extracellular traps [NETs], smooth muscle cell, and monocyte-derived tissue factor) as crucial mediators of the thromboinflammatory processes underlying DVT [4–6].

Ral GTPases, RalA and RalB, are best characterized for regulating exocytosis while exhibiting redundant roles in tumorigenesis and metastasis [7,8]. In platelets, conditional deletion of platelet Ral genes led to a significant defect in P-selectin plasma membrane expression and platelet-leukocyte aggregate formation upon activation [9]. Surprisingly, RalAB double knockout (RalAB DKO) platelets showed specifically a major defect in P-selectin surface expression upon activation while showing normal secretion of soluble α-granule/dense cargo. Other platelet functional responses *in vitro*, arterial thrombosis *in vivo*, and blood cell counts were unaltered.

During DVT initiation, endothelial P-selectin, rather than platelet P-selectin, was shown to be crucial for leukocyte accumulation on the venous wall [6]. Platelets contain significantly more (13-fold) P-selectin than endothelial cells and have a well-established role in leukocyte recruitment/function, while platelet-derived P-selectin is absolutely required for NET formation *in vitro* [10–12]. Despite established procoagulant and proinflammatory roles for P-selectin, a definitive role for platelet-derived P-selectin during DVT *in vivo* remains unclear [13]. Considering the specific secretion defect of platelet P-selectin in RalAB DKO mice, we sought to investigate the role of platelet Ral GTPases in DVT.

## 2 Methods

### 2.1 Materials

Fluorescein isothiocyanate-conjugated CD41 antibody was from Bio-Rad (#MCA2245F); phycoerythrin-conjugated CD45 antibody was from BioLegend (#30-F11); anti-Ly-6G was from Thermo Fisher Scientific (#RB6-8C5); anti–P-selectin antibody was from Santa Cruz Biotechnology (#sc-6942); anti-histone H3 (citrulline R2 + R8 + R17, ChIP grade, #ab5103) was from Abcam. Alexa Fluor-conjugated secondary antibodies were from Thermo Fisher Scientific. DAPI (4′,6-diamidino-2-phenylindole) was from Sigma-Aldrich (#D9542). The Ral GTPase inhibitor RBC8 was from Tocris (#5411). Cross-linked collagen-related peptide (CRP-XL) was custom synthesized by Peptide Synthetics. Unless stated, all other chemicals were purchased from Sigma-Aldrich.

### 2.2 Mice and the flow restriction DVT model

RalA and RalB platelet conditional (Pf4-Cre) RalAB DKO mice were generated as detailed previously [9]. Age- and sex-matched homozygous floxed Pf4-Cre negative littermates were used as controls and denoted wild-type (WT). Male and female mice were bred and maintained at the University of Bristol, and all procedures were in accordance with the United Kingdom Home Office regulations and Animals (Scientific Procedures) Act of 1986 (PPLs 300/3445 and PP5643338). All experiments with animals were conducted in compliance with Animal Research: Reporting of In Vivo Experiments guidelines. DVT was induced in adult mice by partial ligation of the caudal (inferior) vena cava (IVC) as previously described [14]. Side branches were ligated; 0.3 mm stainless steel tubing was used as a spacer. Mice were culled, and IVCs were harvested 24 and 48 hours after surgery, with thrombosis assessed by microscopy and gross pathology.

### 2.3 Ultrasound scanning of blood flow in IVC

Mice were anesthetized with isoflurane (2%, 0.5 L/min), and blood flow in the IVC was visualized before and 30 minutes and 24 hours post-IVC ligation with color Doppler scanning using a Vevo3100 ultrasound imaging system (FUJIFILM VisualSonics) with a high-frequency transducer MX550D.

### 2.4 IVC immunofluorescence microscopy

Fresh tissues were dissected, embedded in optimal cutting temperature, and frozen at −75 °C for sectioning. Tissue sections (5-μm thickness) were fixed with 4% (w/v) formaldehyde/phosphate-buffered saline (PBS) for 30 minutes, permeabilized with 0.2% (v/v) Triton X-100 in PBS containing 1% (w/v) bovine serum albumin for 30 minutes at room temperature (RT) and probed with primary antibodies (1:1000 dilution at 4 °C overnight), following by appropriate Alexa Fluor-conjugated secondary antibodies (1:1000 dilution for 2 hours at RT). Photomicrographs were acquired using a Leica SP5-AOBS DMI6000 inverted epifluorescence microscope.

### 2.5 *In vitro* NETosis assay

Murine neutrophils were isolated from the bone marrow of femurs and tibias of WT mice using Percoll (GE Healthcare Biosciences) gradient centrifugation [15] and seeded (1 × 10^5^ cells/well) in an X-well cell culture chamber (SARSTEDT). Neutrophils were cultured overnight (37°C, 5% CO_2_) in RPMI-1640 growth medium containing 2% (v/v) mouse serum (Sigma-Aldrich) and 0.1 μg/mL recombinant murine G-CSF (PeproTech EC Ltd). Washed mouse platelets were isolated from RalAB DKO mice or their WT littermates, as described previously [9]. For inhibitor-treated samples, platelets (4 × 10^8^/mL) were pretreated with vehicle dimethyl sulfoxide (0.1% v/v) or RBC8 (10 μM) for 1.5 hours at 30 °C to inhibit Ral GTPases, pelleted, and washed 1x in HEPES-Tyrodes solution before activation with CRP-XL (30 μg/mL) for 30 minutes at 30 °C. CRP-XL-activated platelets were then incubated with cultured neutrophils for 16 hours (37 °C, 5% CO_2_). Cells were then washed with ice-cold PBS, fixed with 2% (v/v) paraformaldehyde, and permeabilized with 0.2% (v/v) Triton X-100 in PBS containing 1% (w/v) bovine serum albumin for 30 minutes at RT before incubating with primary antibodies against Ly-6G and citrullinated histone H3 (CitH3; both 1:1000 dilution) at 4 °C overnight. Alexa Fluor-conjugated secondary antibody treatment (1:1000 dilution) was for 2 hours at RT. Cells were then washed with PBS, counterstained with DAPI, and slides were mounted with coverslips. NETosis was quantified as the percentage of CitH3-positive neutrophils using CellProfiler software.

### 2.6 Statistical analysis and colocalization in confocal images

Data were analyzed using GraphPad Prism 8. The presentation of data and appropriate statistical test(s) are described in the figure legends. *In vitro* NETosis data were analyzed with CellProfiler.

## 3 Results and Discussion

**Figure 1:**
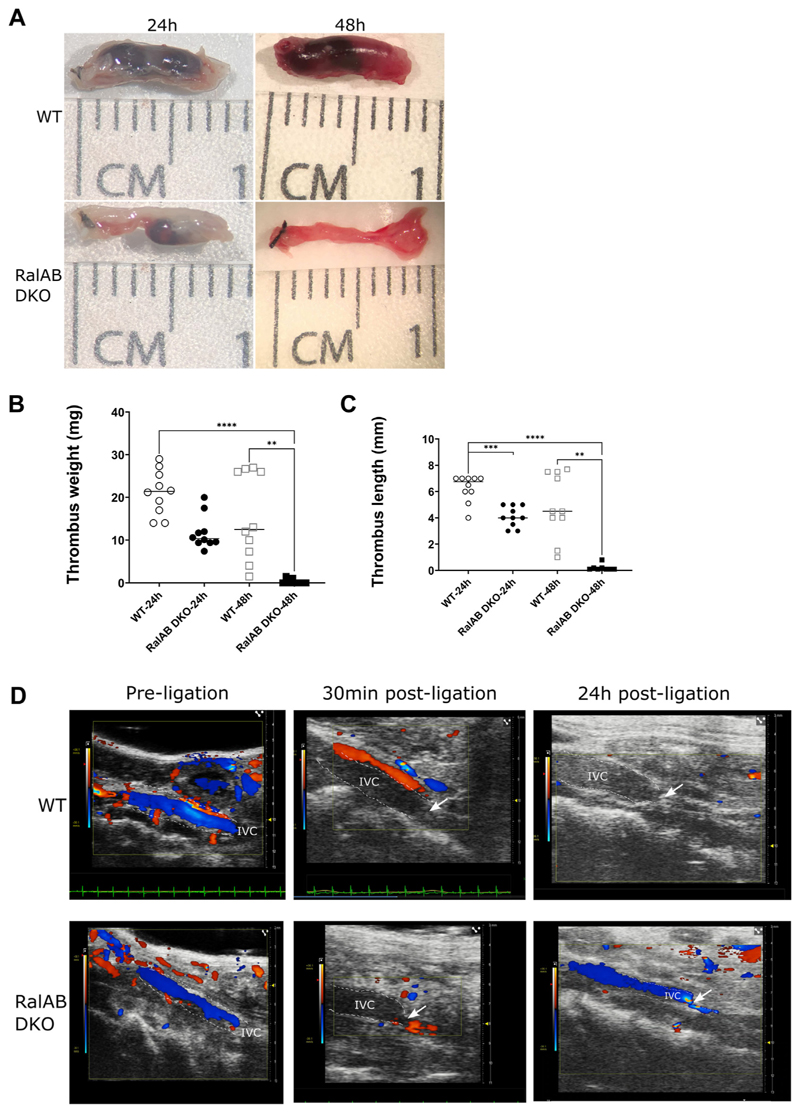
Platelet RalAB GTPases control venous thrombus formation *in vivo*. Wild-type (WT) and RalAB double knockout (RalAB DKO) mice were culled 24 hours or 48 hours post inferior vena cava (IVC) ligation. The IVC was dissected; representative images of dissected IVCs, in which thrombi are contained, are shown in (A). CM, centimeters. Thrombus wet weight (mg; B) and length (mm; C) were measured from IVCs. Each point on the graph represents an independent *n* (*n* = 10 for WT-24h, RalAB DKO-24h, and WT-48h; *n* = 8 for RalAB DKO-48h). The median value is shown by the horizontal lines. A Kruskal–Wallis test with Dunn’s multiple comparisons was performed on (B) and (C) independently. ns, *P* ≥ .05, **P ≤ .01, ****P ≤ .0001. Representative snapshot images from Doppler echo videos of the IVC of WT and RalAB DKO mice prior to ligation and 30 minutes and 24 hours post ligation (D). Blue represents venous flow in the IVC, from right (caudal) to left (proximal), whereas red represents arterial flow, particularly in the neighboring aorta, from left to right. Lighter shades of blue show higher flow velocity. The point of ligation is shown with a white arrow. The approximate location and outline of the IVC is indicated by white dashed lines.

Using a venous stenosis model, we quantified the thrombus formed following surgery [4]. In WT mice, a readily removable red blood cell-rich structure could be found 24 or 48 hours post-IVC flow restriction. In platelet-specific RalAB DKO mice, there was an almost complete absence of DVT at 48 hours after surgery; however, small thrombi were present at 24 hours (Figure 1A–C). Thrombus length and weight, at both 24-hour and 48-hour time points, were significantly reduced in RalAB DKO mice. In parallel experiments, Doppler flow measurements showed that in both WT and platelet RalAB DKO mice, IVC flow was fully blocked at 30 minutes postsurgery (Figure 1D), and venous flow was restored by 24 hours in RalAB DKO mice (Figure 1D) while flow in WT mice was still absent. These data would indicate that Ral GTPases in platelets are important in the stability of the formed thrombi, and the absence of Rals in platelets makes them more fragile and prone to thrombolysis.

For platelet-specific RalAB DKO mice, venous thrombi after 48 hours were absent, although some thrombotic residue in the lumen near the ligature was present, which was firmly adherent to the venous wall (Figure 1A). It is likely that these thrombotic residues were the remains of small thrombi initially formed after blood flow restriction but failed to propagate/stabilize. The RalAB DKO thrombus residues were light pink, indicating low erythrocyte content. Erythrocytes have been shown to interact with VWF and to be recruited by NETs to promote venous thrombus formation under flow restriction [4,16]. Notably, deletion of VWF completely protected mice from venous thrombosis, which was dependent on VWF interaction with platelet GPIbα [4]. Surface GPIbα levels in RalAB DKO platelets are normal (Wersäll and Poole, unpublished data), which, coupled with our observations of unaltered GPIbα-mediated responses in RalAB DKO mice (eg, arterial thrombosis) likely excludes any role for GPIbα-VWF dysfunction in explaining the RalAB DKO DVT phenotype. Venous endothelial P-selectin, rather than platelet P-selectin, is thought to play a critical role in venous thrombosis during blood flow restriction, but immunofluorescence microscopy revealed normal P-selectin expression in the venous endothelium of RalAB DKO mice (data not shown) [5,6].

**Figure 2:**
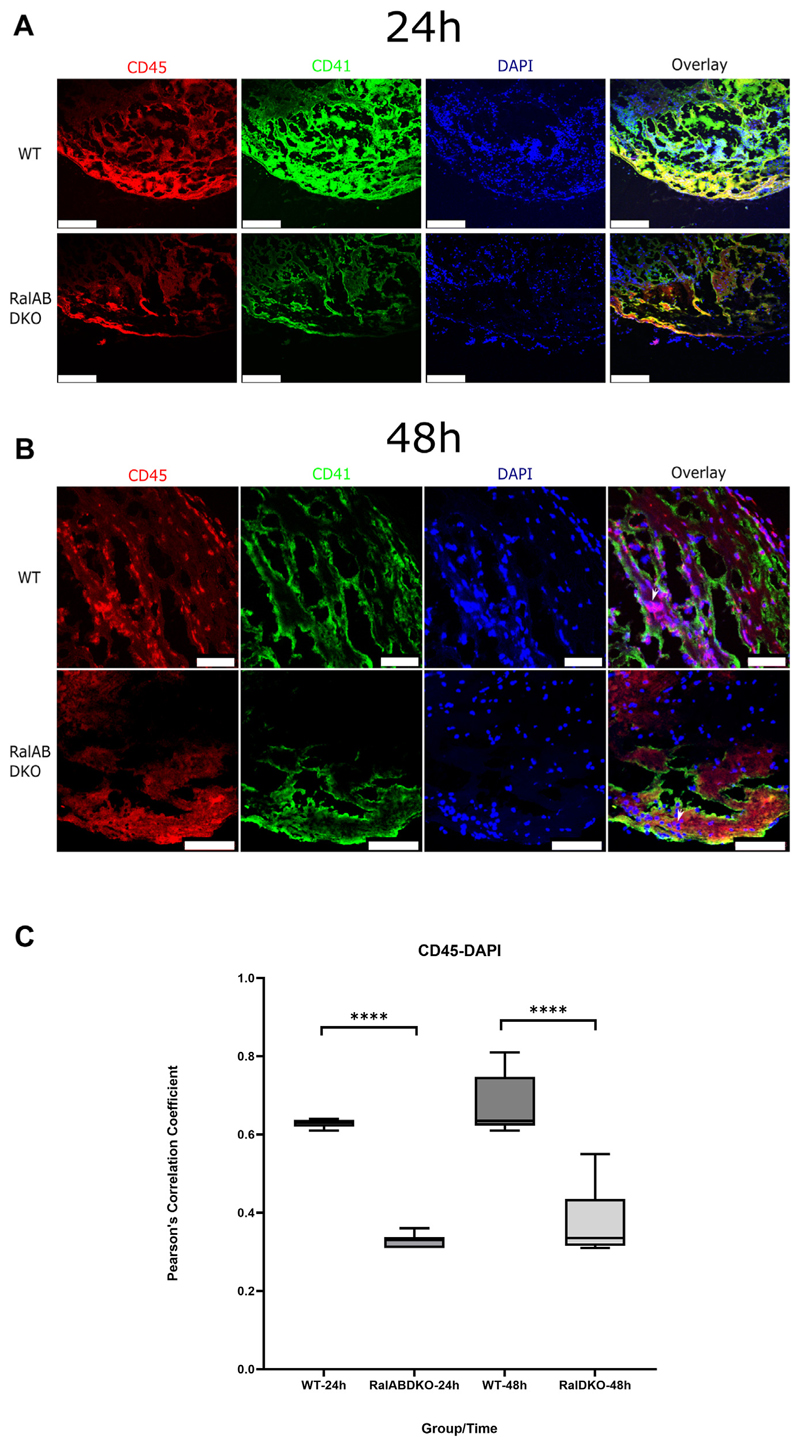
Platelet RalAB double knockout (RalAB DKO) reduces platelet-leucocyte interaction in venous thrombi. Wild-type (WT) and RalAB DKO mice were culled 24 or 48 hours post inferior vena cava ligation. Slices were stained with DAPI (blue), rat anti–CD41-fluorescein isothiocyanate (green), and rabbit anti-CD45 (red) primary antibodies and respective Alexa Fluor-tagged secondary antibody. Z-stack images were acquired using a Leica SP5-AOBS DMI6000 inverted epifluorescent confocal microscope, and maximum projections are presented in the figure. White scale bars are shown for 154.7 μm (A), 50 μm (B, top row), and 75 μm (B, bottom row). Representative images (A and B) of the inferior vena cava cross-sections taken at 24 and 48 hours post ligation, respectively (8 fields of view, 3 mice per condition), and colocalization of CD45 (red) with DAPI (blue) were quantified using the Coloc2 plugin in ImageJ (C). These images highlight the disorganized nuclei in the WT thrombus compared with intact nuclei in the RalAB DKO.

Platelet recruitment is known to support DVT formation *in vivo* [6]. Cross-sections of thrombi, following 24 and 48 hours of blood flow restriction, from WT and RalAB DKO mice were stained with antibodies against CD41 and CD45 to detect recruited platelets and leukocytes, respectively, during DVT. WT thrombi showed a well-organized, scaffolded structure surrounding the midpoint of the cross-section with leukocyte and platelet infiltration into those layers. However, in RalAB DKO thrombi sections, there was no supporting scaffolded structure, with leukocytes and platelets predominantly accumulating at the outer layer (Figure 2A, B). Endothelial P-selectin expression may support this leukocyte and platelet accumulation at the endothelial interface in RalAB DKO mice, but this is not sufficient to propagate a stable venous thrombus. Imaging the scaffolded multilayers in WT thrombi revealed that platelets were lining both sides of the supporting layers, with leukocytes indiltrated in between them (Figure 2A, B). Here, the nuclear stain showed a high degree of colocalization with the leukocyte (CD45) staining (Figure 2C), indicating the nuclei in WT thrombi may have disintegrated and become dispersed through DNA release (NETosis), mixing with intracellular contents, whereas in the RalAB DKO sections, these 2 markers are more distinct and nuclei were mostly intact (Figure 2B). Neutrophils are the major leukocyte component (~70%) in venous thrombi [6], and nuclear lysis and DNA release are hallmarks of NET formation, in common with necrosis and late-stage pyroptosis [17].

**Figure 3:**
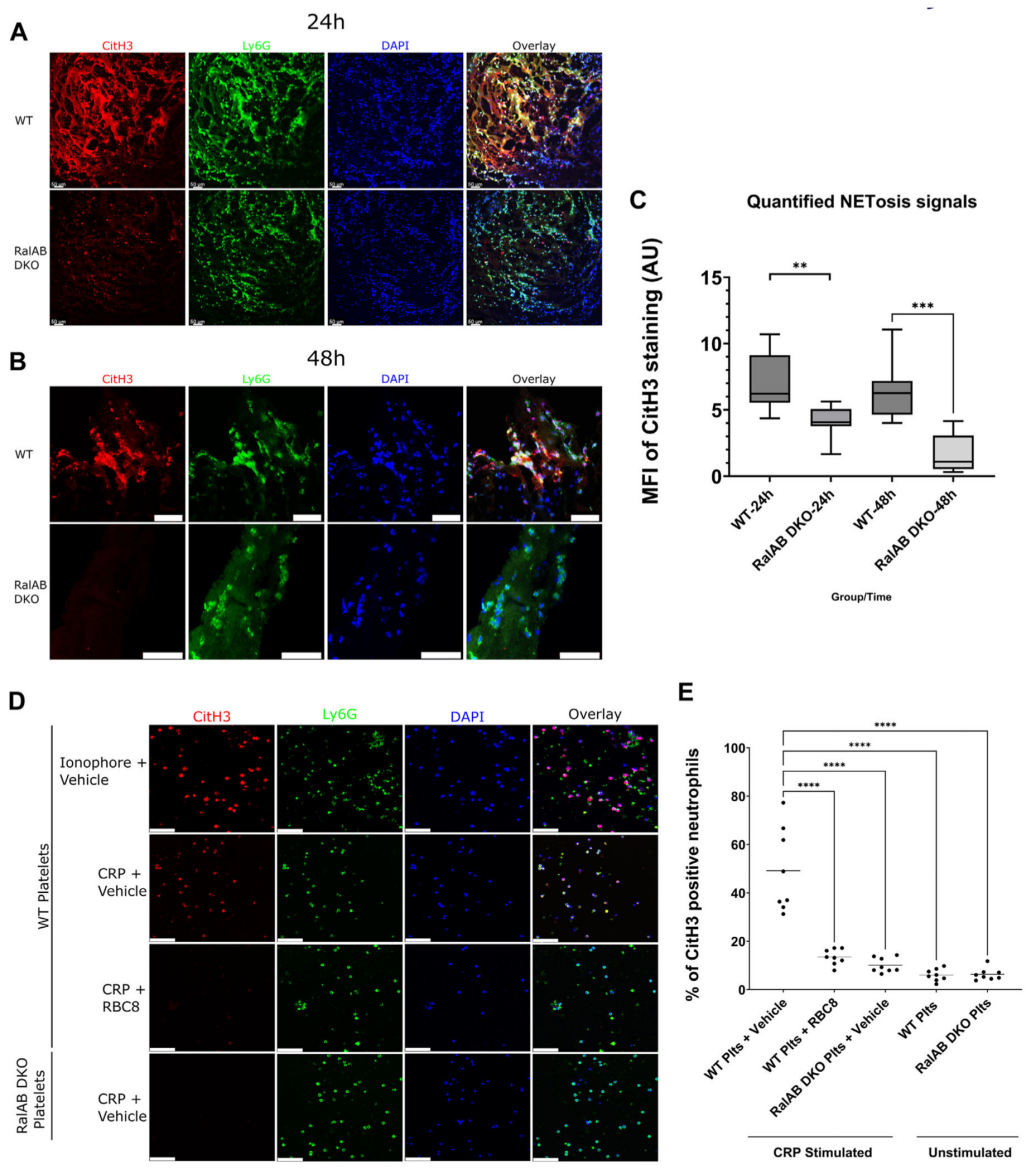
Neutrophil extracellular trap formation is regulated by platelet RalAB *in vitro* and in venous thrombi *in vivo*. Wild-type (WT) and RalAB double knockout (RalAB DKO) mice (A and B) were culled 24 or 48 hours post inferior vena cava (IVC) ligation. The IVC was harvested, and cross-sectional slices were taken prior to fixation and immunofluorescent staining. Samples were stained with DAPI (blue), rat anti-Ly6G (green), and rabbit anti-citrullinated histone H3 (CitH3; red) primary antibodies and respective Alexa Fluor-tagged secondary antibodies. Representative images (A and B) of IVCs harvested after 24 and 48 hours postligation, respectively. The mean fluorescent intensity (MFI) of CitH3 staining (arbitrary units [AU]) was measured, and 2 unpaired *t*-tests were performed to compare genotypes at 24- and 48-hour time points (C). *N* = 7 (of Z-stack maximum projection of 20-40 images of 2 mice of each genotype WT/DKO). **P ≤ .01, ***P ≤ .001. WT mouse neutrophils were isolated and cultured overnight in chamber slides (D) and were then mixed with (i) WT mouse platelets treated with 1 μM A23187 calcium ionophore and vehicle; (ii) WT platelets treated with 30 μg/mL cross-linked collagen-related peptide (CRP-XL) and vehicle; (iii) WT platelets sequentially treated with 10 μM RBC8 and 30 μg/mL CRP-XL; (iv) RalAB DKO mouse platelets treated with 30 μg/mL CRP-XL and vehicle. Samples were fixed and stained using the same fluorescent panel mentioned above. The images presented are representative of 8 independent experiments. All images were acquired using a Leica SP5-AOBS DMI6000 inverted epifluorescent confocal microscope. White scale bars show 50 μm (A and B) and 56.4 μm(D). Neutrophil extracellular trap-positive neutrophils (E; CitH3 and Ly6G expressing) were counted and expressed as a percentage of total neutrophil numbers per field ofview. Data are presented as means ± SE of the means (*n* = 8). A 1-way analysis of variance with Dunnett’s post hoc analysis was performed comparing all groups with WT platelets stimulated with 30 μg/mL CRP-XL and vehicle (****P ≤ .0001).

Platelet-neutrophil interactions, largely facilitated by platelet P-selectin, have been demonstrated to stimulate NET formation *in vitro* and are crucial for DVT propagation [11,18]. To monitor NETs, cross-sections of thrombi from WT and RalAB DKO mice were assessed for the NET marker, CitH3, and neutrophil marker, Ly6G. This revealed a NET signal (CitH3 staining) within the scaffolded thrombi layers in WT mice, indicating that NETosis may have been triggered by platelet-neutrophil interactions. The NET signal was almost absent in RalAB DKO thrombi (Figure 3A–C), suggesting a critical role for platelet Ral GTPases in stimulating NET formation.

To expand on these observations, we studied NET formation following platelet-neutrophil interaction *in vitro*. WT neutrophils were incubated with platelets that had been stimulated with the platelet GPVI receptor agonist CRP-XL. Stimulated WT platelets triggered a profound increase in the number of CitH3 positive neutrophils, with this effect being significantly reduced when RalAB DKO platelets or WT platelets were treated with the Ral inhibitor compound RBC8. These findings confirm the importance of platelet Ral GTPases for NET formation [7] (Figure 3D, E) and may, therefore, suggest that thrombus stability or initiation is dependent on neutrophil NET formation and platelet Ral GTPases. As mentioned, we had previously shown that platelet-leukocyte interactions are substantially attenuated in RalAB deficient platelets, and the substantially diminished platelet P-selectin membrane exposure in RalAB DKO platelets is not due to altered kinetics of release or change in surface membrane shedding of P-selectin [9].

**Figure 4:**
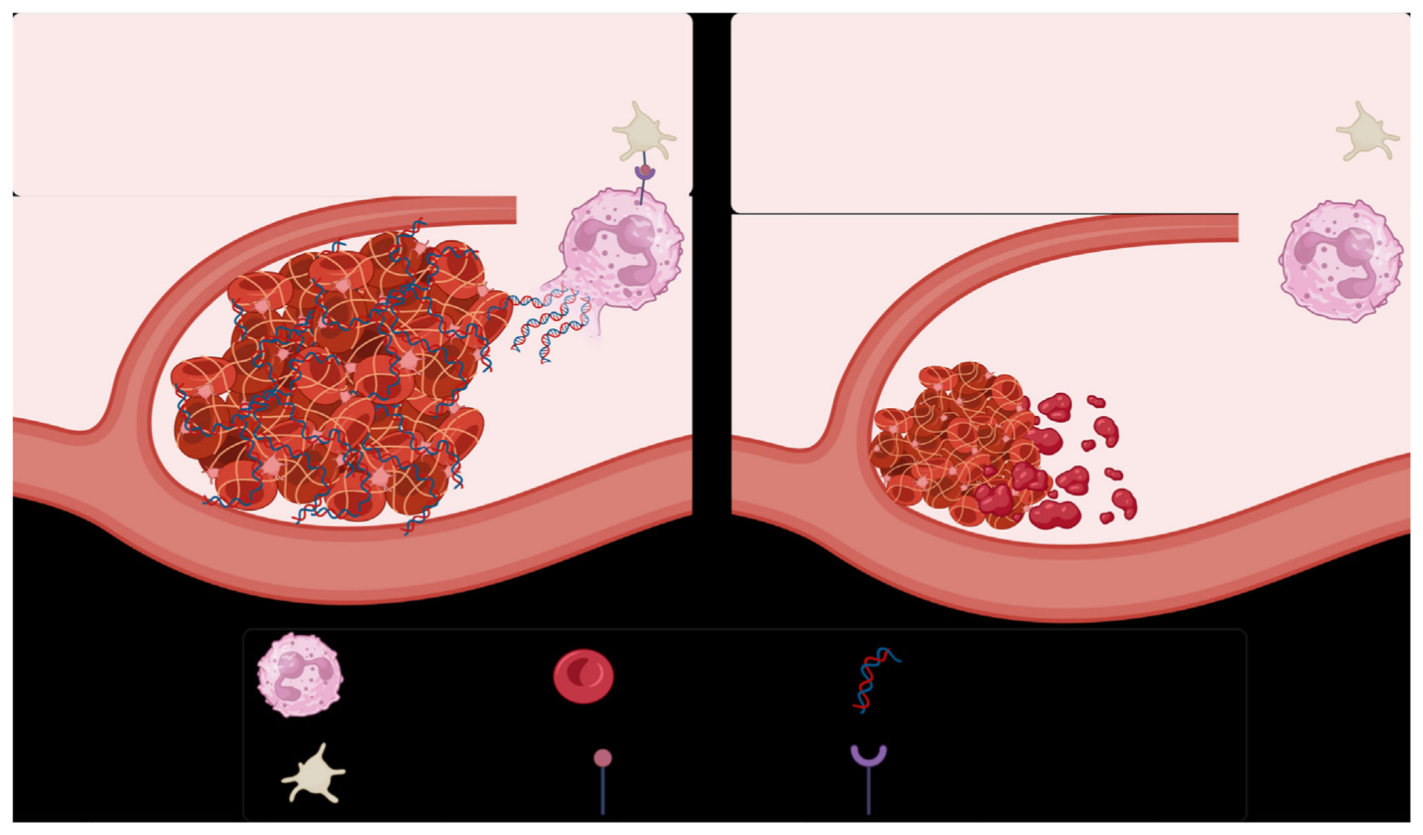
RalAB double knockout in platelets reduces thrombus formation and stability. Activated wild-type platelets express surface P-selectin and interact with P-selectin glycoprotein ligand-1 on neutrophils, inducing neutrophil NETosis. Neutrophil extracellular traps stabilize a thrombus, increasing its longevity. Activated RalAB double knockout platelets do not present P-selectin. Thus, NETosis and subsequent thrombus stabilization do not ensue. Created using Biorender.com.

In conclusion, platelet Ral GTPases may have a significant role in DVT initiation under flow restriction via their regulation of NET formation (Figure 4). While a definitive molecular mechanism for this observation is yet to be determined, platelet P-selectin has been shown previously to promote NETosis [11] and, in the DVT setting, is a platelet RalAB-dependent fashion. The absence of Ral GTPases in platelets leads to smaller and more fragile venous thrombi formed. It is possible that platelet Ral GTPases could be therapeutic targets to control venous thrombotic disease. Small molecule inhibitors of Ral GTPases, which are currently under preclinical investigation as possible cancer therapies, could therefore be beneficial in the treatment or prophylaxis of DVT.
